# Supplementary material for: Investigation into the Direct Photolysis Process of Photo-Induced RAFT Polymerization by ESR Spin Trapping
Source: Polymers (Basel). 2019 Oct 21;11(10):1722. doi: 10.3390/polym11101722 (PMC6835659; doi:10.3390/polym11101722)
Supplement: Supplementary file 1 [file polymers-11-01722-s001.pdf]

Supporting Information For

Investigation into the direct photolysis process of photo-induced reversible addition fragmentation chain transfer (RAFT) polymerization by electron spin resonance (ESR) spin trapping

Jiajia Li [1], Mengmeng Zhang [1], Jian Zhu [1,*] and Xiulin Zhu[1,2]

[1] State and Local Joint Engineering Laboratory for Novel Functional Polymeric Materials, Jiangsu Key Laboratory of Advanced Functional Polymer Design and Application, Department of Polymer Science and Engineering, College of Chemistry, Chemical Engineering and Materials Science, Soochow University, Suzhou 215123, China; 20154209023@stu.suda.edu.cn (J.L.); 20184209174 (M.Z.); xlzhu@suda.edu.cn (X.Z.)

[2] Global Institute of Software Technology, No 5. Qingshan Road, Suzhou National Hi-Tech District, Suzhou 215163, China

* Correspondence: chemzhujian@suda.edu.cn (J.Z.); Tel.: +86-512-65880726 (J.Z.)

Additional Results

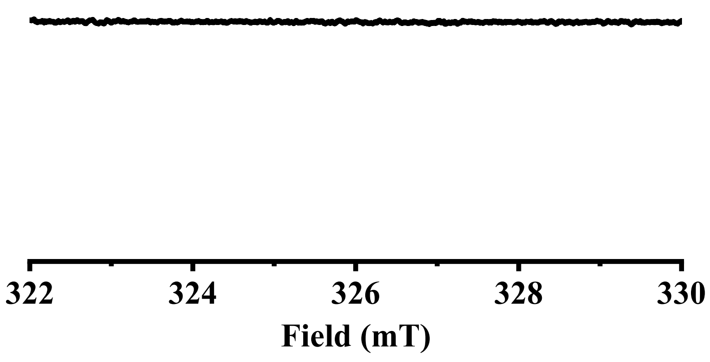

Figure S1. X-band ESR spectra of EXEP in toluene under light with λ_{max} = 440 nm taken at t = 3 min, $[EXEP]_0$ = 0.225 mol L^{-1}.

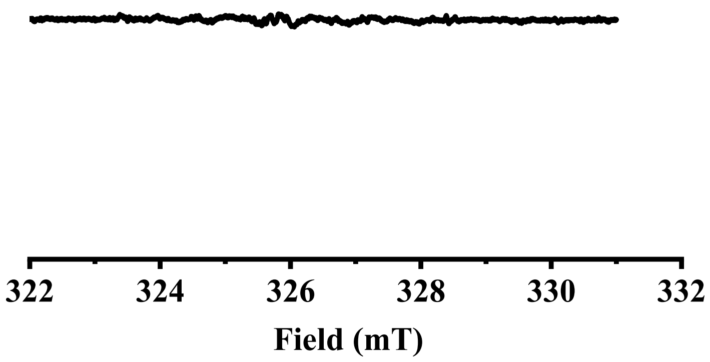

Figure S2. X-band ESR spectra of DMPO in toluene under light with λ_{max} = 440 nm taken at t = 3 min, $[DMPO]_0$ = 0.675 mol L^{-1}.
